# Statement of Retraction: Pristimerin inhibits neuronal inflammation and protects cognitive function in mice with sepsis-induced brain injuries by regulating PI3K/Akt signalling

**DOI:** 10.1080/13880209.2024.2323353

**Published:** 2024-03-05

We, the authors, Editors and Publisher of the journal *Pharmaceutical Biology*, have retracted the following article:

Weimin Xue, Yaqiang Li & Mei Zhang (2021) Pristimerin inhibits neuronal inflammation and protects cognitive function in mice with sepsis-induced brain injuries by regulating PI3K/Akt signalling, Pharmaceutical Biology, 59:1, 1349-1356, https://doi.org/10.1080/13880209.2021.1981399

Since publication, the authors identified that the results shown in Figure 4 cannot be repeated.

As this directly impacts the reproducibility and validity of the reported results and conclusions, the authors alerted the issue to the Editor and Publisher.

During the investigation by the journal, additional concerns were raised such as:

1. Similarity in Figure 6 with Figure 6 in the following article published in *Frontiers in Neurology*:
  - Auti ST and Kulkarni YA (2019) Neuroprotective Effect of Cardamom Oil Against Aluminum Induced Neurotoxicity in Rats. Front. Neurol. 10:399. https://doi.org/10.3389/fneur.2019.00399
2. Similarity in Figure 5 with figures in the following articles:
  - Figure 8 in Sun, X., Gan, L., Li, N. et al. Tabersonine ameliorates osteoblast apoptosis in rats with dexamethasone-induced osteoporosis by regulating the Nrf2/ROS/Bax signalling pathway. AMB Expr 10, 165 (2020). https://doi.org/10.1186/s13568-020-01098-0
  - Figure 4a in Liu, X, Wang, B. Fuscoside Attenuates Bone Loss in Bone Defects by Regulating The Rankl/Nlrp3/Opg Pathway in Rats. Cell Journal 23 (2021), 451-456. https://doi.org/10.22074/cellj.2021.7736
  - Figure 6 in Jiang, T., You, H., You, D. et al. RETRACTED ARTICLE: A miR-1275 mimic protects myocardiocyte apoptosis by regulating the Wnt/NF-κB pathway in a rat model of myocardial ischemia–reperfusion-induced myocardial injury. Mol Cell Biochem 466, 129–137 (2020). https://doi.org/10.1007/s11010-020-03695-w
  - Figure 3 in Fang, X, Zhou, H., at al. MiR-1906 attenuates neuropathic pain in rats by regulating the TLR4/mTOR/ Akt signaling pathway. Translational Neuroscience 10 (2019), 175-179. https://doi.org/10.1515/tnsci-2019-0031

Whilst the authors have fully cooperated with the investigation, when they were asked for an explanation for the similarities for these figures, they were unable to address the concerns raised as they were unable to provide the raw data.

In light of the concerns about the integrity of the reported results, all parties have agreed to retract the article to ensure correction of the scholarly record.

We have been informed in our decision-making by our policy on publishing ethics and integrity and the COPE guidelines on retractions.

The retracted article will remain online to maintain the scholarly record, but it will be digitally watermarked on each page as “Retracted”.

